# The Problem of Tuberculosis

**Published:** 1916-04-22

**Authors:** 


### THE PROBLEM OF TUBERCULOSIS.

### The Patient, the Nurse, and Questions of Economy.

It is not a little difficult to get people to differentiate in their mind's eye between the tuberculosis problem and the tuberculous patient. It is even more difficult to get intelligent persons to see that sometimes the tuberculosis problem, if properly handled, must claim a prior interest over the personal comfort of the consumptive individual. The wholesale segregation of the advanced and infectious cases of pulmonary tuberculosis is, of course, too far in advance of public opinion to be entertained as a practical measure, but that is not to say that a stricter control over such cases in relation to their own immediate or family environment cannot justifiably be enforced.

Several factors have been credited with influencing the decline in tuberculous disease which is apparently still continuing, though at a diminished rate. Some authorities are convinced that the key to the whole problem lies in the management of the advanced infectious case; others see in improving housing and better feeding a lessening risk of infection and an increasing resistance. Whatever view one may prefer to take, one cannot get away from the fundamental fact that the bacillus of tuberculosis is the fountain of evil. The increasing of the strength of our armour is surely a poor way of defending ourselves, if it is possible to deprive the enemy of ammunition. The problem, therefore, resolves itself into a consideration of the practicability of materially diminishing the supply of tubercle bacilli without interfering too widely with the rights and liberties of patients and their nearest relatives.

A study of what are known as the "family histories" of large numbers of consumptives forces upon us the conclusion that infection transmitted from one member of a household to another is a very potent factor in the dissemination of the disease. On account of its chronicity the administrative difficulties of dealing with pulmonary tuberculosis are of quite a different order from those arising in connection with other infectious diseases. The "treatment" of consumption in sanatoria is beset with difficulties, and it is a question whether the permanent benefit to the community bears a reasonable proportion to the large expense involved. It is only fair to state, what critics too often forget, that sanatoria are required not only to deal with unpromising material and even quite advanced cases, but are now sometimes hampered by considerations of a character more political than clinical.

It becomes therefore all the more urgent to consider whether or no it would be more profitable to divert some of the money now spent upon the maintenance of sanatoria from this purpose to means directed to lessening the infective power of a patient in his own home. There is much to be said for this line of action, and the difficulties of organisation, when closely examined, are not so formidable as might be imagined. It is not suggested that the really early case should be treated anywhere else than in a sanatorium, nor that the principle of institutional segregation should not be pushed as far as practicable. Sanatorium treatment costs somewhere about thirty shillings a week per patient, and unfortunately in no small proportion of cases the amount expended is not at all commensurate with the benefit accruing to the patient's health or with the amount of temporary protection to the community consequent on the absence of the patient from home or workplace.

The advocates of the policy of looking very closely after patients in their houses would rely largely upon the activities of specially trained tuberculosis nurses, and would devote the funds at their disposal to paying an increased staff of such nurses, to providing prophylactic apparatus, additional sleeping accommodation and possibly supplementary nourishment for other members of the family, if not for the patient himself. In the conduct of anti-tuberculosis campaigns the tendency has been perceptibly to enlarge the sphere of the visiting nurse, and when our national economic resources again permit us to use funds for such purposes, there is little doubt but that the nurse will find even greater demands made upon her. This "policing," as it may be termed, of the infectious consumptive is, of course, only a substitute for isolation; the cheapest way of attacking the bacillus is probably to enforce segregation of the advanced cases as was suggested by Newsholme. For the present this is only likely to be carried out on a small scale.

To turn out an efficient tuberculosis nurse it is necessary, firstly, to get hold of the right sort of woman; secondly, to offer her thorough and practical training; and thirdly, to make her realise in the widest sense her responsibilities in regard to the tuberculosis problem as distinct from those towards the tuberculous patient.

There has recently appeared a book devoted to the functions and qualifications of the tuberculosis nurse* which takes a broader view of the training and the duties of this important officer than any of the now somewhat numerous manuals intended for her instruction. The authoress herself has been engaged for eight years in special tuberculosis work, first as "field" nurse of the Visiting Nurse Association of Baltimore, later as organiser and director of the Tuberculosis Division of the Baltimore Health Department. She has, therefore, had the useful experience of seeing the work advance through the struggling stages of private enterprise into the well-organised grooves of the city machinery. The fact that the material in the pages of her well-composed book was gathered in Baltimore need not deter English readers from making use of the excellent teaching therein, for the conditions indicated or dealt with are common to all towns and cities, and

* *The Tuberculosis Nurse: Her Functions and Qualifications.* A Handbook for Practical Workers in the Tuberculosis Campaign. By Ellen N. La Motte. (New York and London: G. P. Putnam's Sons. 1915. 6s.)

her ideas and principles are fully applicable to our own country. Her experience points to the conclusion alluded to above, that the most direct way of controlling tuberculosis is through the segregation (though she adds voluntary) of the distributor. The chief and foremost duty of the public health nurse is to assist in removing the patient from an environment where he is dangerous to one where he is harmless—all other duties are subsidiary to this. In this respect the authoress is supported by Dr. Louis Hamman, of the Phipps Tuberculosis Dispensary, who has written an introductory chapter which is a valuable essay on the general aspect of the tuberculosis problem.

Miss La Motte's book deals systematically chapter by chapter with all the phases of tuberculosis work, keeping always in view the work of the nurse as a public health servant. She recognises the fact that physicians have their limitations and shortcomings, and that the nurse not infrequently has diplomatically to minimise the results of doctors' indifference and even mistakes. An excellent chapter is the one on the nurse in relation to the physician. We suspect that not all our general practitioners would subscribe to everything in this chapter, but in the near future such points as may now be taken exception to will be accepted without demur. The nurse will be recognised as no longer the handmaiden of the doctor, but as a co-worker, and, as the authoress aptly remarks, "it is always the badly trained physician who fears the well-trained nurse." Some of the difficulties referred to in connection with the exploitation of patients by practitioners are fortunately so rare in this country that a good deal of the matter in this section will come as a surprise to our own nurses.

Nevertheless obstructionists will occasionally be met with in "the greatest profession, the meanest of trades," as Dr. Cabot called medicine. On the other hand, the nurse is largely instrumental in unearthing new cases and bringing them under medical care. The daily work of the nurse is well described, and a typical office with its arrangements for records and reports is dissected with instructive detail. On the subject of visiting and getting into touch with retiring dispositions the book gives that truly helpful advice which can only be given by those who have had the requisite experience. It need hardly be said that such matters as come under the headings of actual nursing of phthisical patients, the hygiene of the home, and the distribution and use of prophylactic supplies are dealt with from the same practical point of view. In fine, this book is distinctly one which should be read by nurses, and even medical officers, specialising in tuberculosis work. In its contents there is much which should be taken to heart by those who are attempting to further the anti-tuberculosis campaign.

